# Compatibility Polymorphism Based on Long-Term Host-Parasite Relationships: Cross Talking Between *Biomphalaria glabrata* and the Trematode *Schistosoma mansoni* From Endemic Areas in Brazil

**DOI:** 10.3389/fimmu.2019.00328

**Published:** 2019-04-09

**Authors:** Mariana G. Lima, Lângia C. Montresor, Joana Pontes, Ronaldo de C. Augusto, Jairo Pinheiro da Silva, Silvana C. Thiengo

**Affiliations:** ^1^Curso de Pós-Graduação em Ciências Veterinárias, Universidade Federal Rural do Rio de Janeiro, Seropédica, Brazil; ^2^Laboratório de Referência Nacional em Esquistossomose–Malacologia, Instituto Oswaldo Cruz/FIOCRUZ, Rio de Janeiro, Brazil; ^3^Área de Biofísica, Departamento de Ciências Fisiológicas, Instituto de Biologia, Universidade Federal Rural do Rio de Janeiro, Seropédica, Brazil; ^4^UMR 5244 Univ Perpignan via Domitia-CNRS-IFREMER-Univ Montpellier, Interactions Hôtes-Pathògenes-Environnements (IHPE), Université de Perpignan via Domitia, Perpignan, France

**Keywords:** snail-trematode, host-parasite, interaction, compatible, incompatible, infectivity, resistance, schistosomiasis

## Abstract

Sympatric snail populations have been kept in the laboratory since the isolation of the parasite from the field. To evaluate the influence of the intermediate host in the infectivity of *S. mansoni*, this allopatric strain was compared to two sympatric strains, from different geographical origins, and with different time of maintenance in the laboratory. Snail–trematode compatibility was accessed for a total of nine possible combinations (three snail populations, three schistosome strains), using different charges of parasite: 1, 5, 10, and 15 miracidia/snail. Each *S. mansoni* strain was characterized according to its infectivity phenotype that reflects the efficiency of their infection mechanism and all *B. glabrata* populations were characterized according to its (in)compatible phenotype that reflects the level of (un)susceptibility they display. For all host-parasite combinations tested the dose-response relation indicated a trend for an increase in the infectivity of *S. mansoni* when higher miracidial doses were used. SmRES-2 presented the highest overall infectivity rate, especially in the SmRES-2/BgRES interaction with 15 miracidia/snail. However, SmRES was more infective to BgBAR than SmRES-2, indicating that SmRES strain was more infective at the first contact with this new host than after 2 years of interaction (SmRES-2). BgBAR presented the highest susceptibility to infection. SmRES and SmRES-2 are the same parasite strains. It seems that during these 2 years of interaction, BgBAR acted like a filter and shifted the compatibility polymorphism of the strain SmRES. SmRES-2 became more infective to BgRES (sympatric) than to BgBAR (allopatric), and conversely, SmRES was more infective to BgBAR (allopatric) than to BgRES (sympatric). This interplay suggests that epigenetic mechanisms are prompting these changes. This study concerns with infection of *B. glabrata* snails from different Brazilian localities with *S. mansoni* in allopatric and sympatric associations that will partially help in understanding the natural epidemiology of schistosomiasis within natural snail populations in watercourses. This work demonstrates that there is a shift on the compatibility polymorphism profile resulting from sympatric and allopatric interactions of *B. glabrata* and *S. mansoni* that constantly change during the time of interaction.

## Introduction

Schistosomiasis is caused by the trematode *Schistosoma mansoni* Sambon, 1907 and occurs in the Neotropical region, Africa and the Middle East. According to the World Health Organization—WHO (1) ~240 million people are affected by schistosomiasis in the world and more than 700 million live in areas at risk (2). *Biomphalaria glabrata* is the main intermediate host of *S. mansoni*, the agent of intestinal Schistosomiasis, in South America (3).

The snails are obligate intermediate hosts of *S. mansoni* and they are necessary for the larval development and for the transmission of the parasite to vertebrates. The dynamics underlying this process includes genetic and epigenetic mechanisms related to the interaction between parasites and their hosts. Parasites with heteroxenous life cycles require a number of hosts to complete their development, and each host act as a potential filter, modulating both the infection intensity and the genotypes/phenotypes that reach the subsequent host (4–6). Consequently, in a complex life cycle, intermediate hosts may potentially affect the transmission of parasite genotypes and their expressed phenotypes (7).

Many studies on schistosomiasis are carried out using parasites and hosts from cycles maintained in the laboratory. Several factors exert influence on the host parasite relationships, and consequently, on the results of laboratory trials (8). To better understand the host-parasite systems used for research purposes, some aspects must be considered (i.e., *S. mansoni* strains may be maintained in the laboratory in allopatric or in sympatric snails; the time of interaction in the laboratory can vary from some months to several decades). The compatibility of *S. mansoni* and *B. glabrata* depends at least on the age of the snail; former interactions between snail-schistosome; the genetic profile of both parasite and host; and the environment (9).

In summary, there is a global picture in this host-parasite interaction, and an infectivity-susceptibility mosaic shows a co-evolutionary dynamic known as compatibility polymorphism (10, 11). In recent years, integrative approaches, including large scale molecular analysis, have been used and resulted in an increase on our understanding on the mechanisms underlying resistance/compatibility (11). The research community, especially in the last three decades, used laboratory-maintained cycles to investigate host-parasite relationships, so that is the importance to understand how these interactions works.

*Biomphalaria glabrata* is the main intermediate host of *S. mansoni* in South America and its compatibility varies among different populations and individuals. This interaction was chosen as a model, and concentrated research efforts have been carried out over the years (9). Different *B. glabrata* laboratory populations presents different degrees of susceptibility to a strain of *S. mansoni* and different *S. mansoni* strains presents different levels of infectivity to a snail population (12–15). The success or failure of infection reflects a complex interplay between the host's defense mechanism and the parasite's infective strategies. Furthermore, compatibility is strain specific, and one parasite strain that is highly infective to a snail population could be less or un-infective to another. Likewise, one snail population that presents high susceptibility to one strain of schistosome could be partially or completely unsusceptible to another (15–18).

Usually, one laboratory keeps the cycle of *S. mansoni*, and the biological material they produce is donated for different researchers and institutions. Over the last 4 years, our laboratory donated more than 200 times *B. glabrata* and *S. mansoni* samples. The Malacology Laboratory of the Oswaldo Cruz Institute is a National Reference laboratory for Schistosomiasis-Malacology (LRNEM) and thus receives samples of snails from all over the country. More than 80 populations of *Biomphalaria* snails are kept in the LABMAL, most of them comprising the species that are intermediate hosts in South America, *Biomphalaria glabrata, Biomphalaria straminea*, and *Biomphalaria tenagophila* (Table 1). Eight strains of *S. mansoni* are also maintained using sympatric snails and albino Swiss-Webster mice. Some strains have been in the laboratory for more than 30 years, and other for <2 years.

**Table 1:** List of *Biomphalaria glabrata, Biomphalaria tenagophila*, and *Biomphalaria straminea* populations maintained and inbred at Laboratory of Malacology—FIOCRUZ/RJ.

	**Species**	**Locality**	**Country**		**Species**	**Locality**	**Country**
1	*Biomphalaria glabrata*	Vila de cura/Venezuela	VEN	40	*Biomphalaria tenagophila*	São José dos Campos—SP	BRA
2	*Biomphalaria glabrata*	Barreiro—MG	BRA	44	*Biomphalaria tenagophila*	São José dos Campos—SP	BRA
3	*Biomphalaria glabrata*	Dique do Tororó—Salvador—BA	BRA	45	*Biomphalaria tenagophila*	Itamaraju—BA	BRA
4	*Biomphalaria glabrata*	Doresópolis—MG	BRA	46	*Biomphalaria tenagophila*	Asunción (pigmentado)/Paraguai	PAR
5	*Biomphalaria glabrata*	Pedrão—BA	BRA	47	*Biomphalaria tenagophila*	Taubaté (albino)—SP	BRA
6	*Biomphalaria glabrata*	Alagoinhas—BA	BRA	48	*Biomphalaria tenagophila*	Taubaté (pigmentado)—SP	BRA
7	*Biomphalaria glabrata*	Núcleo Bandeirantes—DF	BRA	49	*Biomphalaria tenagophila*	UHE—Simplício-MG	BRA
8	*Biomphalaria glabrata*	São Sebastião do Passé—BA	BRA	50	*Biomphalaria tenagophila*	Alto da Boa Vista (albino)—RJ	BRA
9	*Biomphalaria glabrata*	Coração de Maria—BA	BRA	51	*Biomphalaria tenagophila*	Jacarepaguá—RJ	BRA
10	*Biomphalaria glabrata*	São Sebastião do Passé—BA	BRA	52	*Biomphalaria tenagophila*	Joinville (albino)—SC	BRA
11	*Biomphalaria glabrata*	Curitiba—PR	BRA	53	*Biomphalaria tenagophila*	Joinville (pigmentado)—SC	BRA
12	*Biomphalaria glabrata*	Esteio—BA	BRA	54	*Biomphalaria tenagophila*	Andorinhas—ES	BRA
13	*Biomphalaria glabrata*	Medina/Venezuela	VEN	55	*Biomphalaria tenagophila*	Posadas/Argentina	ARG
14	*Biomphalaria glabrata*	Pontezinha—PE	BRA	56	*Biomphalaria tenagophila*	Taim—RS	BRA
15	*Biomphalaria glabrata*	Barreiro—Belo Horizonte—MG	BRA	57	*Biomphalaria tenagophila*	Ayui/Argentina	ARG
16	*Biomphalaria glabrata*	Ressaca—Belo Horizonte—MG	BRA	58	*Biomphalaria tenagophila*	Corrientes—San Roque	ARG
17	*Biomphalaria glabrata*	Ressaca—Belo Horizonte—MG	BRA	59	*Biomphalaria tenagophila*	Rio Marinho—ES	BRA
18	*Biomphalaria glabrata*	Jacobina—BA	BRA	60	*Biomphalaria tenagophila*	Joana D'arc—ES	BRA
19	*Biomphalaria glabrata*	Irara—BA	BRA	61	*Biomphalaria tenagophila*	Piraí—RJ	BRA
20	*Biomphalaria glabrata*	Touros—RN	BRA	62	*Biomphalaria tenagophila*	São José dos Campos—SP	BRA
21	*Biomphalaria glabrata*	Belém—PA	BRA	63	*Biomphalaria tenagophila*	São José dos Campos—SP	BRA
22	*Biomphalaria glabrata*	Varzea—BA	BRA	64	*Biomphalaria tenagophila*	Itamaraju—BA	BRA
23	*Biomphalaria glabrata*	Saúde—BA	BRA	65	*Biomphalaria tenagophila*	Asunción (pigmentado)/Paraguai	PAR
24	*Biomphalaria glabrata*	Viseu—PA	BRA	66	*Biomphalaria tenagophila*	Taubaté (albino)—SP	BRA
25	*Biomphalaria glabrata*	Guadaloupe	GUA	67	*Biomphalaria tenagophila*	Taubaté (pigmentado)—SP	BRA
26	*Biomphalaria glabrata*	Paulista—PB	BRA	68	*Biomphalaria straminea*	Tangará—SP	BRA
27	*Biomphalaria glabrata*	Belen—Las Tinajitas/Venezuela	VEN	69	*Biomphalaria straminea*	Picos—RN	BRA
28	*Biomphalaria glabrata*	Belém—PA	BRA	70	*Biomphalaria straminea*	Guapimirim—RJ	BRA
29	*Biomphalaria glabrata*	Bragança—PA	BRA	71	*Biomphalaria straminea*	Jaguarari—RN	BRA
30	*Biomphalaria glabrata*	Santa Luzia (albino)—MG	BRA	72	*Biomphalaria straminea*	Itapagipe—MG	BRA
31	*Biomphalaria glabrata*	Teolândia—BA	BRA	73	*Biomphalaria straminea*	Belém—PA	BRA
32	*Biomphalaria glabrata*	Coroatá—MA	BRA	74	*Biomphalaria straminea*	Jabuticabas—MG	BRA
33	*Biomphalaria glabrata*	Alhandra—PB	BRA	75	*Biomphalaria straminea*	Tangará—RN	BRA
34	*Biomphalaria glabrata*	Nisia Floresta—RN	BRA	76	*Biomphalaria straminea*	Picos—PI	BRA
35	*Biomphalaria glabrata*	Sr. do Bonfim—BA	BRA	77	*Biomphalaria straminea*	Guapimirim—RJ	BRA
36	*Biomphalaria glabrata*	Capitão Andrade—MG	BRA	78	*Biomphalaria straminea*	Jaguarari—RN	BRA
37	*Biomphalaria glabrata*	Catú—BA	BRA	79	*Biomphalaria straminea*	Itapagipe—MG	BRA
38	*Biomphalaria tenagophila*	Curitiba—PR	BRA	80	*Biomphalaria straminea*	Belém—PA	BRA
39	*Biomphalaria tenagophila*	Asunción (albino)/Paraguai	PAR	81	*Biomphalaria straminea*	Jabuticabas—MG	BRA

LABMAL is one of the most important laboratories of schistosomiasis in Brazil, with the greatest alive stock of *B. glabrata* and *S. mansoni*. The laboratory produces and donates a huge number of samples for different research purposes, i.e., drugs target on schistosomiasis treatment and control (19) and to study proteins that significantly and distinctively influenced DNA transactions in *S. mansoni* (20). We strongly believe that investigating and understanding the interaction between them will improve the quality of research. Among the eight main strains that are kept in the lab, the strain from Ressaca (MG) is the most donated one. Over the last years a decrease of compatibility between snail (BgRES) and schistosome (SmRES) from Ressaca has been observed. An empirical attempt has been made to deal with this problem. Part of the Ressaca strain was shifted to an allopatric snail, from Barreiro (MG). Along 2 years of allopatric interaction, an increase in the infection rate of SmRES was observed. This observation lead us to believe that epigenetic mechanisms are involved and rise out this hypothesis to explain some of them and encourage new research directions.

Moreover, these results prompted us to look further and to question the features that might be related to different profiles of host-parasite-environment interaction that generates different profiles of (in)compatibility: time of interaction (laboratory-bred population), geographic origins, genetic diversity, etc. Hereupon, the phenomena observed in our laboratory could be a trace of dynamic genetic interaction, changing through long/short-term- interaction and epigenetic mechanisms. These results also rise questions on the consequence of these changes (if they occur) to the immune-physiological profile related to the interaction. In summary, this study is the very first step in the attempt toward understanding the processes underling these interactions using “omics” approaches and epigenetic analysis.

In the present work, we investigate the multi-parasite strain susceptibility of *B. glabrata* stocks and conversely, the multi host strain infectivity of *S. mansoni* stocks based on the host-parasite interaction time. And that is just the first step to understand the variation of compatibility amongst the main strains produced in the LABMAL that will allow the development of better research strategies, improving the quality of laboratory research on schistosomiasis.

## Material and Methods

### *Biomphalaria glabrata* Populations

Sympatric snail populations were kept in the laboratory since the isolation of the parasite from the field. These sympatric populations, BgRES, from Ressaca neighborhood, Contage (Minas Gerais State, Southeast Brazil), and BgTEO, from Teolândia (Bahia State, Northeast Brazil), were used to maintain the cycle of their respective *S. mansoni* strain. Another population, BgBAR, from Barreiro neighborhood, Belo Horizonte (Minas Gerais State, Southeast Brazil) has been used for 2 years to keep the *S. mansoni* strain from Ressaca, representing an allopatric association.

### *Schistosoma mansoni* Strains

Two *S. mansoni* strains, selected according to the time of parasite-host interaction within the laboratory, has been kept in laboratory in sympatric snails and in mice (Swiss-Webster) for 8 and 30 years: (i) SmRES is the oldest strain, and was isolated in the 1980's from Ressaca neighborhood, Minas Gerais State, Brazil; (ii) SmTEO is the youngest strain kept in sympatric snails, and was isolated from Teolândia municipality, Bahia state, Brazil in 2010's. One allopatric association has been kept for 2 years in the laboratory using allopatric snails (BgBAR) to maintain the cycle of SmRES. Thereafter we will refer to this parasite strain as SmRES-2.

It is important to highlight that SmRES-2 does not infect BgRES before this study and, since the allopatric association was produced (2 years ago, in the laboratory), SmRES-2 infected only BgBAR snails.

### Matrices and Maintenance of Snails

Ten adult snails of each strain (matrices) were placed in transparent glass aquarium filled with 1,500 ml of dechlorinated water and 0.5 g of CaCO3. They were kept in a room with temperature (25°C), luminosity (*photoperiod* of 12 h in white light) and humidity (70% RH). Weekly, on alternate days, the aquarium was cleaned, and the snails fed with fresh leaves of lettuce (*Lactuca sativa* L.) *ad libitum*. The F_1_ generation of each matrix was used for the compatibility tests.

### Compatibility Tests

**Figure 1:**
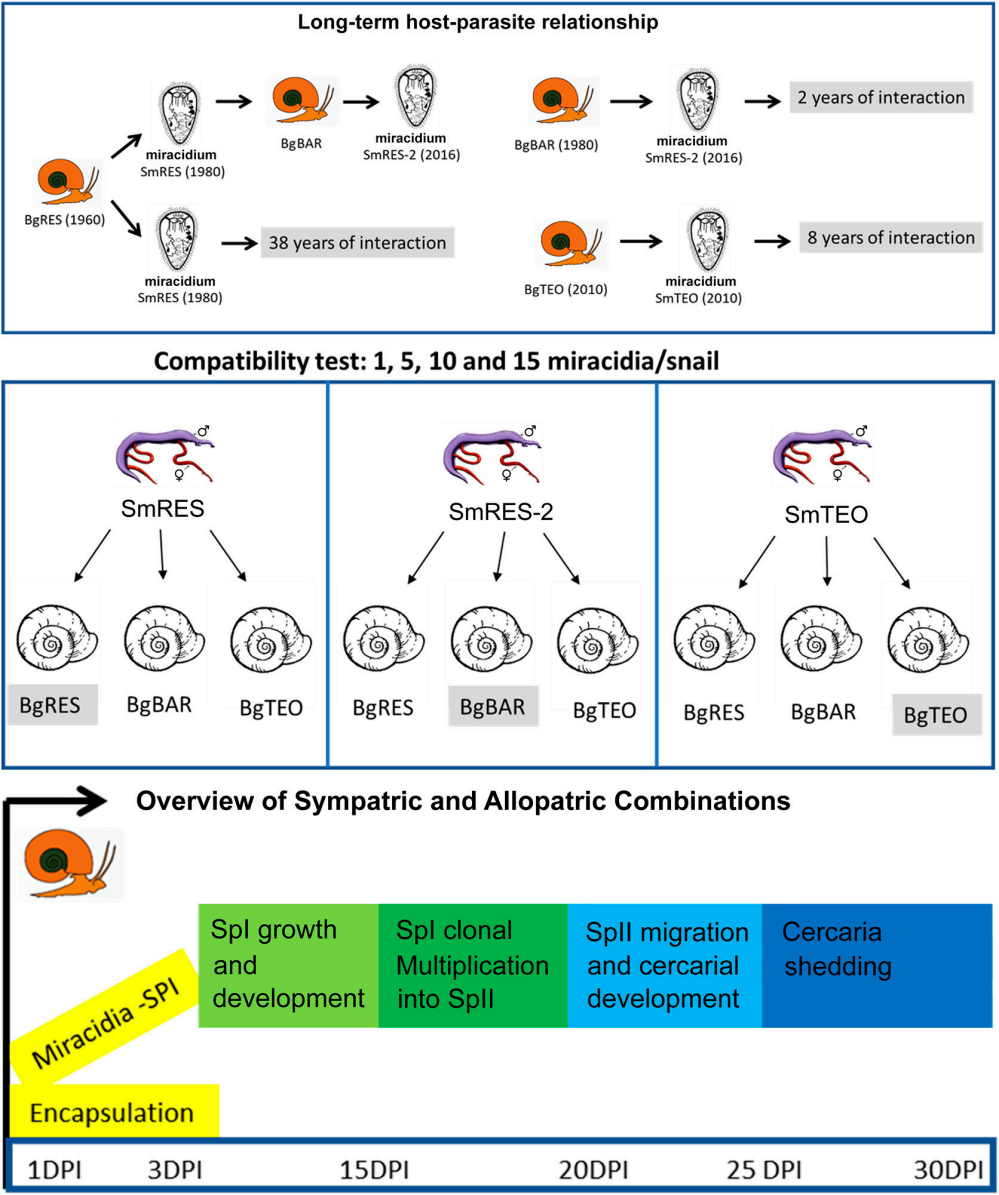
Overview of experimental procedures. Long-term host-parasite relationship: *Biomphalaria glabrata* (Bg) and *Schistosoma mansoni* (Sm) stocks at Laboratory of Malacology, BgRES and SmRES from Ressaca–MG collected in 1980', 38 years of host-parasite interactions; BgBAR form Barreiro–MG collected in 1980', 2 years of host-parasite interactions with SmRES-2; BgTEO and SmTEO from Teolândia–BA collected in 2010', 8 years of host-parasite interactions. Compatibility test with 1, 5, 10, and 15 miracidia: Nine host-parasite combinations were established: BgRES/SmRES; BgBAR/SmRES, BgTEO/SmRES, BgBAR/SmRES-2, BgRES/SmRES-2; BgTEO/SmRES-2; BgRES/SmTEO; BgBAR/SmTEO and BgTEO/SmTEO. Overview of Sympatric and Allopatric combinations: After exposition, molluscs were placed in aquaria and kept under the same maintenance's condition of the matrices until 15 days post-infection (DPI). The snails were fixed in modified Railliet-Henry solution (21) for the observation of primary sporocysts (SpI).

To evaluate the infection rate, the studies were conducted based on different doses of parasites for a dose-response correlation. Individual infections were carried out using 1, 5, 10, and 15 miracidia/snail. Other doses were tested (30, 50 and 100 miracidia/snail), however, they caused 100% mortality. Nine host-parasite combinations were tested: BgRES/SmRES; BgBAR/SmRES, BgTEO/SmRES, BgBAR/SmRES-2, BgRES/SmRES-2; BgTEO/SmRES-2; BgRES/SmTEO; BgBAR/SmTEO, and BgTEO/SmTEO as described in Figure 1.

Ten snails of each population (6–9 mm) were individually exposed for 4 h to different doses of freshly hatched miracidia in 20 ml glass containers containing 5 ml dechlorinated water, and after the exposure time they were observed under a stereomicroscope to count miracidia that did not penetrate during the exposure period.

After exposure, the snails were placed in aquaria and kept under the same matrices conditions until 15 days post-infection. Thereafter, they were removed from the aquaria, placed individually in 10 ml of 1% Hypnol anesthetic solution for 4 h to relax the tissues. Then they were immersed in water heated to 70°C for 45″, then immersed in water at room temperature, and with the aid of tweezers, the region of the headfoot mass was pulled with a gentle pull to detach the insert from the columellar muscle. The snails were fixed in modified Railliet-Henry solution (21) for the observation of primary sporocysts (SpI).

For all experimental groups, the establishment of infection was determined by the presence of primary sporocysts in the tissues. The SpI number was determined after thorough dissection of each exposed snail, including deeper tissues. The degree of compatibility was quantified by the proportion of infected snails and the infection intensity was established according to the number of SpI by infected snails (15).

The results were analyzed using ANOVA and Tukey *post-hoc* test (R version 3.4.4) and are presented in graphs. Differences were considered statistically significant when *P* < 0.05. The R Script (Data Sheet 1) and the data analysis are provided in the Supplementary Table 1.

## Results

### Infectivity of *Schistosoma mansoni* Strains

**Figure 2:**
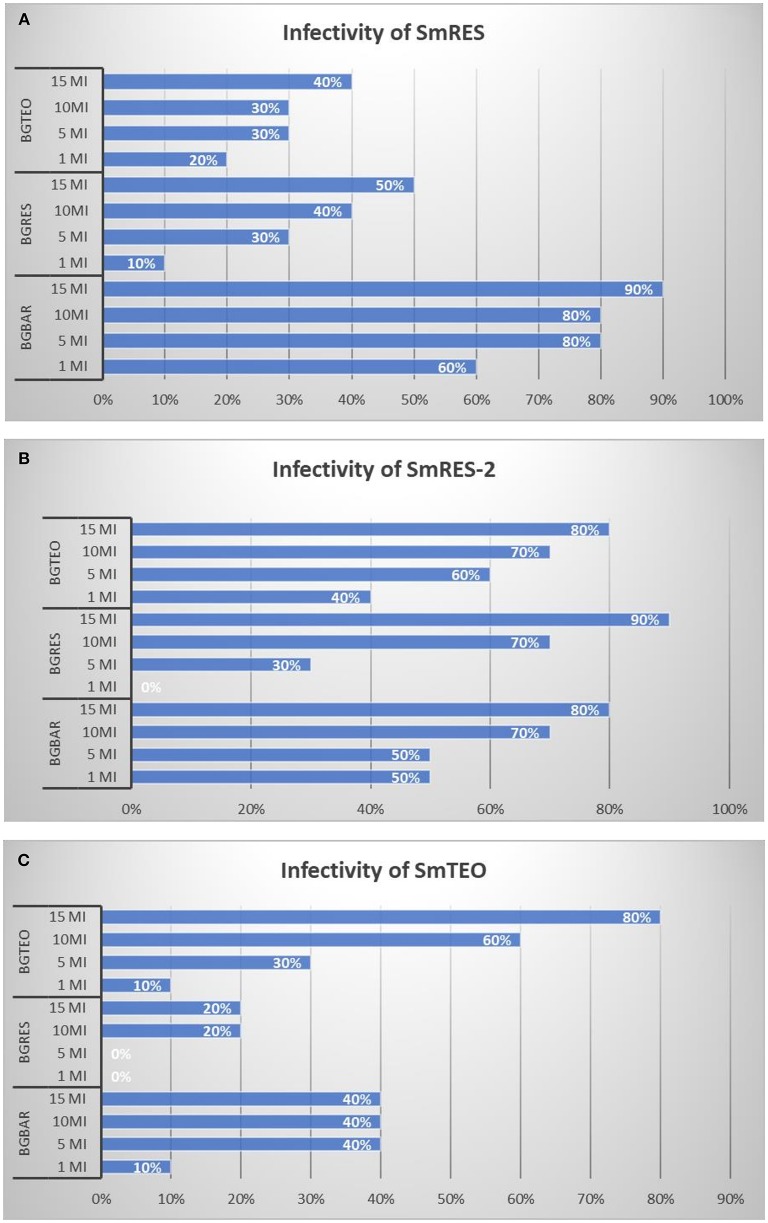
Infectivity of *Schistosoma mansoni* strains (%) SmRES **(A)**, SmRES-2 **(B)**, and SmTEO **(C)** in the different *Biomphalaria glabrata* strains (BgRES, BgBAR, and BgTEO) exposed to different doses of miracidia (Mi).

For all host-parasite combinations tested the dose-response relation indicated a trend for an increase in the infectivity of *S. mansoni* when higher miracidial doses were used (Figures 2A–C). The strain SmRES presented low infectivity to the sympatric snails (10–50%) and to the allopatric snails BgTEO (20–40%) (Figure 2A). Furthermore, when interacting to another allopatric snail, the BgBAR, this strain presented high infectivity rates (60–90%).

The strain kept in allopatric snails, SmRES-2, presented the highest infectivity on all *B. glabrata* populations tested. This strain showed 40–80% of infectivity to BgTEO, 0–90% to BgRES, and 50–80% to BgBAR (Figure 2B). The SmTEO strain presented low infectivity to allopatric snails (0–20% BgRES; 10–40% BgBAR). The lowest infectivity was seen in the interaction SmTEO/BgRES, which presented 0% infectivity at 1 and 5 miracidia/snail and 20% at 10 and 15 miracidia/snail (Figure 2C). However, the infectivity to sympatric snails was high (10–80%) (Figure 2C).

It is important to highlight that the strain SmRES, which is maintained in laboratory using sympatric snails, presented low infectivity to this snails, and high infectivity to the allopatric BgBAR snails (Figure 2A). On the other hand, after 2 years of interaction with allopatric snails (BgBAR), the strain (renamed BgRES-2) became highly infective to the sympatric snails (BgREs) and to the allopatric snail BgTEO. Thus, the 2 years of interaction between SmRES-2 and BgBAR resulted in an increase on the infectivity of the strain to both sympatric and allopatric hosts (BgRES and BgTEO).

### Susceptibility of *Biomphalaria glabrata* Populations

**Figure 3:**
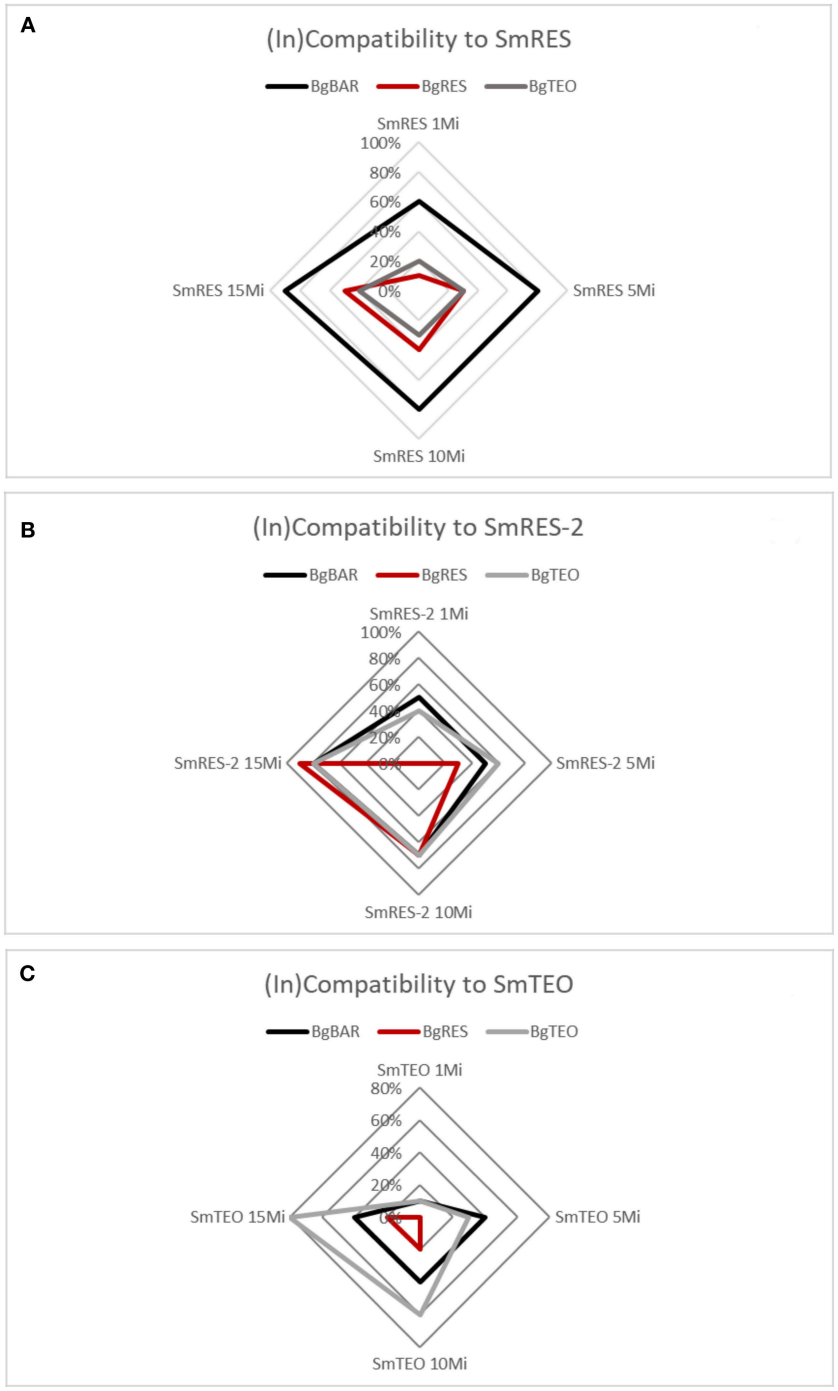
(In)Compatibility of *Schistosoma mansoni* strains SmRES **(A)**, SmRES-2 **(B)**, and SmTEO **(C)** to *Biomphalaria glabrata* strains (BgRES, BgBAR, and BgTEO) at different doses of miracidia (Mi).

From the host's perspective, the susceptibility varies according to the strain of *S. mansoni* (Figures 3A–C). The BgBAR population is far more susceptible to SmRES than BgRES and BgTEO (Figure 3A). When exposed to the higher miracidial doses (10 and 15 miracidia), all populations were highly susceptible to SmRES-2 (Figure 3B). On the other side, the interaction between BgBAR and SmTEO showed the lowest compatibility among all combinations tested, while BgTEO presented high susceptibility to its sympatric strain, especially at higher miracidial doses (Figure 3C). It is important to note that after 2 years of allopatric interaction with SmRES-2, BgBAR presented lower susceptibility to the renamed strain (Figure 3B) when compared to SmRES strain, which was kept in sympatric snails (Figure 3A). On the other hand, BgRES presented higher susceptibility to the sympatric strain after it was kept for 2 years in allopatric interaction with BgBAR (renamed SmRES-2) (Figures 3A,B).

### Infection Intensity

**Figure 4:**
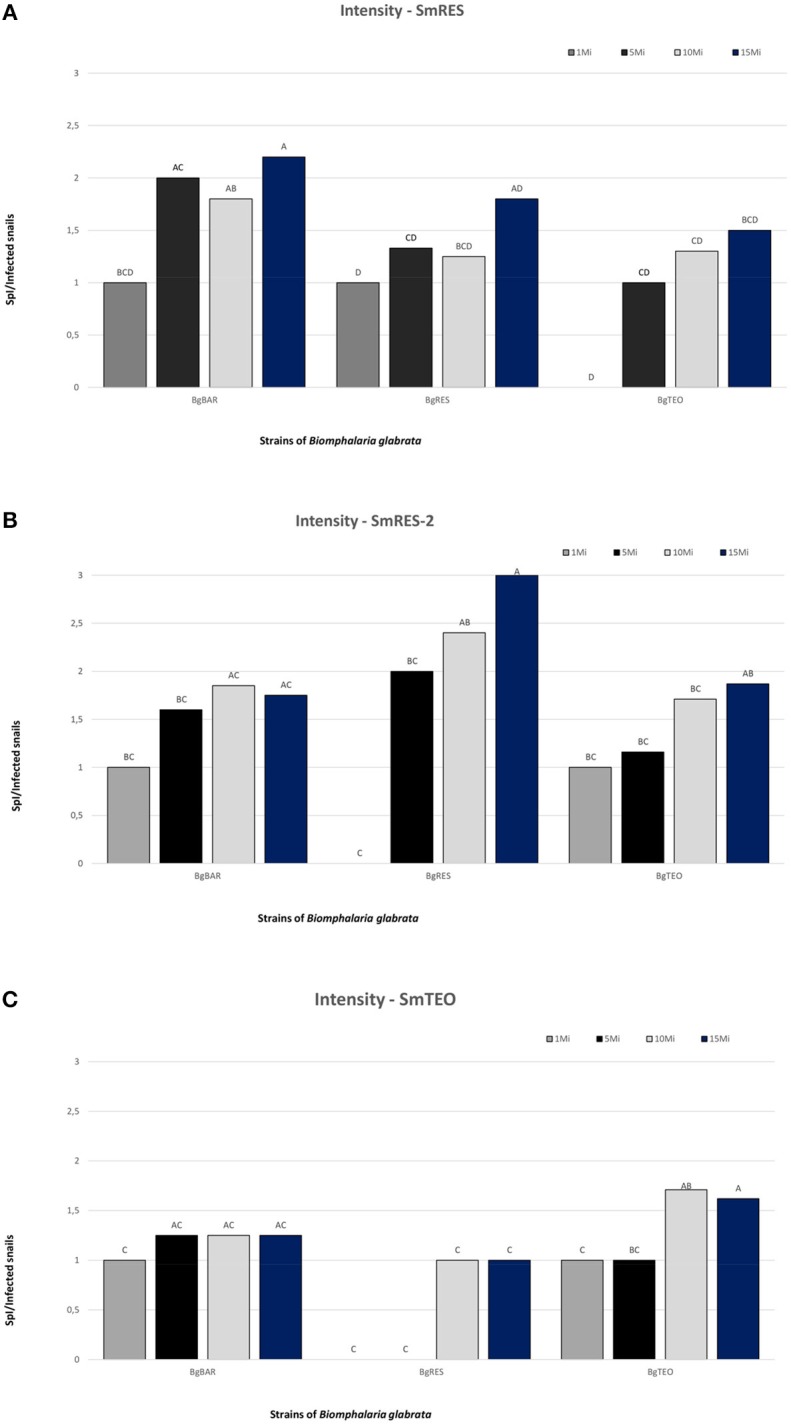
Intensity of infection of the different miracidial doses of *Schistosoma mansoni* SmRES **(A)**, SmRES-2 **(B)**, and SmTEO **(C)** in the different strains of *Biomphalaria glabrata* (BgRES, BgBAR, and BgTEO). Different letters means significant difference.

The intensity of the infection is given by the ratio: number of SpI/number of infected snails. For all host-parasite combinations, there was a trend for an increase in the intensity of the infection when higher miracidial doses were used. For comparison purposes, we will consider as “low infection intensity” the interactions that do not exceeded 1.5 SpI/Infected snail at any one of the tested doses (SmRES/BgTEO; SmTEO/BgBAR; SmTEO/BgRES). Most of the interactions that presented higher infection intensity (Figures 4A–C) were those that also presented high infectivity rates (Figures 3A–C): SmRES-2/BgRES; SmRES-2/BgBAR; SmRES-2/BgTEO; SmRES/BgBAR. The only exception was SmRES/BgRES that, despite the low infectivity rate (10–50%), presented high infection intensity. The infection intensity was higher in sympatric combinations for SmRES-2 and SmTEO, however, for SmRES, the infection intensity was higher in an allopatric combination (SmRES/BgBAR).

The strain SmRES-2 presented the highest infection intensity, especially in the SmRES-2/BgRES sympatric interactions with 10 and 15 miracidia/snail. All interactions with this strain presented high infection intensity (Figure 4B). The interactions with the strain SmRES were the second most intense, and only one interaction, SmRES/BgTEO, presented low infection intensity (Figure 4A). SmRES/BgBAR with 15 miracidia/snail (mean = 2.2 SpI/snail) was the second most intense interaction (Figure 4A). The interactions with the strain SmTEO were the least intense and the allopatric interactions presented low infection intensity: SmTEO/BgBAR; SmTEO/BgRES (Figure 4C). The highest infection intensity for SmTEO was recorded in the sympatric interaction with BgTEO, at 10 and 15 miracidia/snail.

The profile of development of SpI depends on the schistosome strain, snail' population and also on the dose used in the infection. The strains SmRES and SmRES-2 showed interesting results concerning the host-parasite compatibility. Despite the different designation, SmRES and SmRES-2, represents the same strain. The only difference between them is the population of *B. glabrata* used in their maintenance. The strain SmRES has been kept in sympatric BgRES snails since its isolation, 38 years ago. The strain SmRES-2 represents an allopatric association, in which BgBAR snails were used, for 2 years, to keep SmRES strain. This intermediate host shift (from BgRES to BgBAR) generated changes on the strain compatibility polymorphism. The original SmRES strain is less susceptible to BgRES than to BgBAR, while SmRES-2 is more susceptible to BgRES than to BgBAR. Regarding BgTEO, this snail was the least compatible to both strains; however, SmRES-2 is more compatible to this snail population than SmRES. Thus, comparing the intensity of the infection of these strains, it is possible to infer that BgBAR snails provoked some changes on the compatibility polymorphism of SmRES-2 strain: I- increase in the compatibility with the sympatric BgRES population; II- increase in the compatibility with the allopatric BgTEO population; III- decrease in the compatibility with the allopatric BgBAR population that has been used to keep the cycle for 2 years.

Data analysis showed that SmRES-2 is the strain with the highest percentage of miracidia transformation into SpI, with 90% of infection in SmRES-2/BgRES at 15 miracidia/snail. The rate of transformed SmRES miracidia was higher in the SmRES/BgBAR allopatric interaction (2.2 transformation at 15 miracidia/snail) than in the SmRES/BgRES sympatric interaction (1.8 transformation at 15 miracidia/snail) (Figures 4A,B).

**Figure 5:**
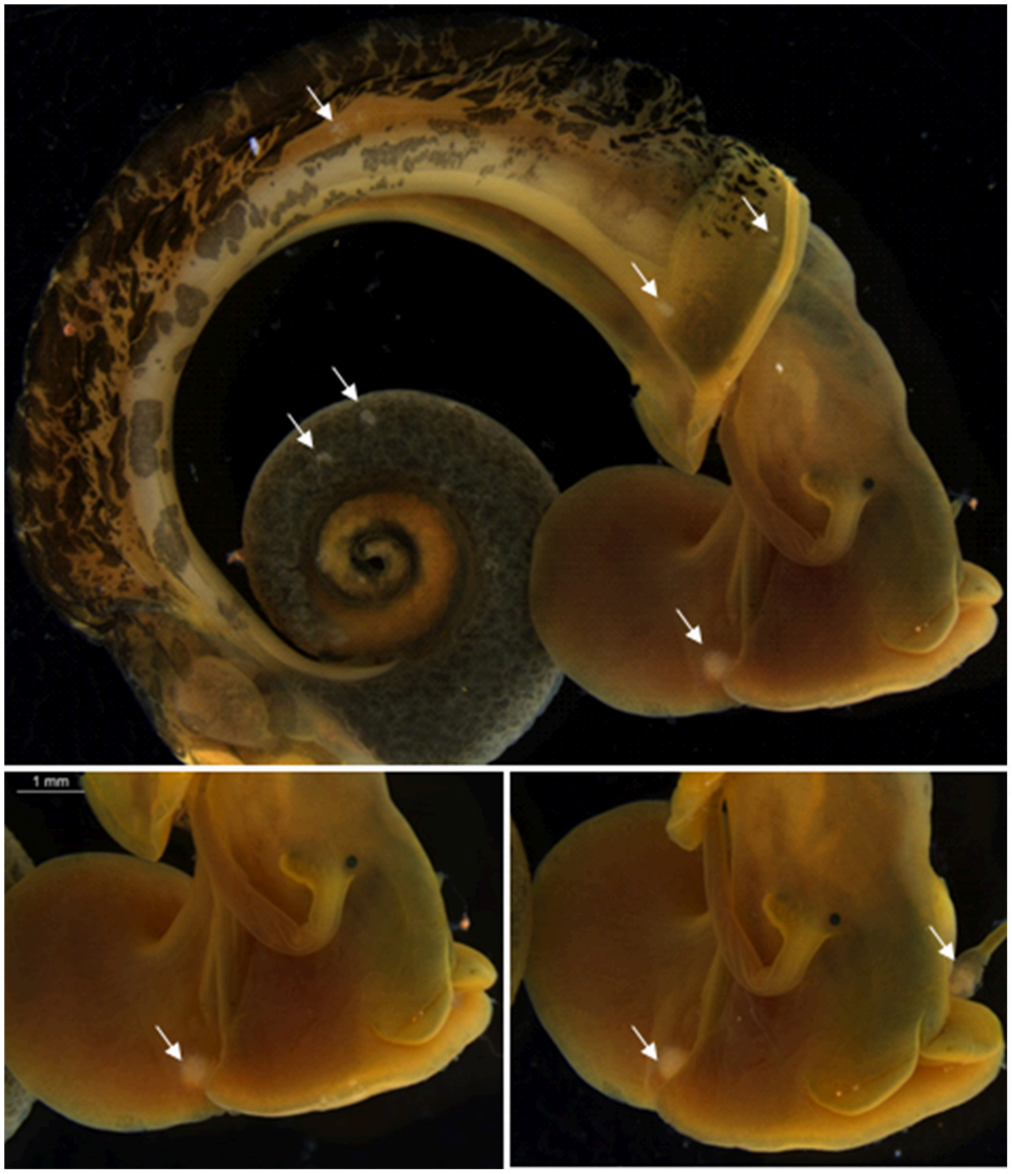
Sporocysts I of *Schistosoma mansoni* (SmRES-2) in *Biomphalaria glabrata*, from Ressaca (BgRES), at a dose of 15 Mi (Scale bar = 1 mm). Arrow = SpI (primary sporocyst).

Figure 5 shows SmRES-2 miracidia transformed into SpI in a BgRES snail.

## Discussion

Compatibility studies have been mainly carried out with laboratory cycles maintained for one or several generations, therefore the data are probably poorly representative of the diversity in original populations. However, to address these limitations, it is extremely necessary to start from some point; thus, host/parasite compatibility has been approached in this study using sympatric and allopatric combinations of miracidia and snails. The compatibility between snails and schistosomes reflects the sum of molecular and environmental determinants, which are variable within and between populations. This complex system not only involve the host's defense mechanisms and the parasite's infective strategies (17, 22, 23) but also the matched or mismatched status of the host and parasite phenotype (9, 18).

In Brazil, only a few laboratories have laboratory-bred *B. glabrata* and biological cycles of *S. mansoni* under certified conditions and comply with strict maintenance rules. Among them, the LABMAL maintains limnic and land snails and that acts as gold standard for other research centers inside and outside the country.

Our investigation on snail/schistosome compatibility was based on long-term interactions between parasite and host. We emphasize that all the *B. glabrata, S. mansoni* strains used in this study were maintained in the laboratory during several years (from 8 to 38), and their susceptibility and infectivity profiles are not representative of the corresponding natural populations. Thus, our alive stock of snails and parasites is inevitably different from wild stocks, nevertheless, they are very useful for studies on the host-parasite relationships, especially on “omics” approaches that may help us to understand these relationships on natural populations. Our study was guided by the previous study done by Theron et al. (15) who addressed another component of these relationships when they extent the susceptibility/un-susceptibility level of different populations of *B. glabrata* confronted to the same panel of different *S. mansoni* strains. According to the authors, this cross talking between hosts' and parasites' strains make possible to characterize its “multi-parasite susceptibility phenotype” and its “multi-host infectivity phenotype,” respectively. Without a doubt, the results obtained in our study showed that host and parasite strains present marked differences in their capacity to resist or to infect.

From the parasite's perspective, marked variations on the infectivity spectrum were observed and varied from the poorly infective SmTEO to the highly infective SmRES-2, which was able to infect more than 80% of the three hosts tested. It is interesting to observe that, among sympatric combinations, SmRES/BgRES and SmTEO/BgTEO displayed low levels of compatibility. According to the pattern of the dose-response curve, a gradient could be established. Such phenomenon could be linked to a mechanism recently described by Galinier et al. (10). These authors state that in the multistep infectious interactions of parasites and hosts, a genetically explicit model reveals that polymorphism is greater at recognition loci than at effector loci, and that host-genotype and parasite-genotype interactions are greater at the recognition phase than at the effector phase (10). Moreover, the high incompatibility between BgRES/SmRES may be a result of a genetic mismatch. On the other hand, the high compatibility between the sympatric combination SmRES-2/BgRES seems to be a consequence of the maintenance of this strain in allopatric BgBAR snails and may indicate that BgBAR acted as a filter. In fact, after its establishment in BgBAR snails, SmRES-2 became highly infective to BgRES. However, after 2 years, there was a decrease in the degree of infectivity of SmRES-2 to BgBAR, when compared to the infectivity of SmRES to BgBAR. These finds indicate that when a strain is maintained in the laboratory, there is a loss of compatibility along the time. These finds will be further investigated in the second phase of this study, where we will increase the number of samples and use proteomic and transcriptomic approaches.

*Schistosoma mansoni* and *B. glabrata* from Ressaca are one of the oldest combinations established in the laboratory. Data collected during these years of routine maintenance of both host and parasite strains show meaningful change in their compatibility along the time. This fact prompted us to investigate this phenomenon: Why does the compatibility level decreases during successive laboratory generations? It is important to highlight that BgRES/SmRES are the most used and donated samples, and this shift was the red flag that demonstrated the importance to understand this interaction and also to compare to the other strains.

In order to understand the *S. mansoni/B. glabrata'* interaction and do not lose the SmRES strain, the lab began to use BgBAR to keep the cycle of SmRES; changing the sympatric combination to an allopatric combination. After 2 years of interaction we observed that BgRES became less susceptible meanwhile BgBAR was more compatible. This phenomenon leads us to investigate the multi-panel of *B. glabrata-S. mansoni* crosstalk. Our results demonstrate that after 2 years of interaction with BgBAR, the strain SmRES, now named as SmRES-2 is more effective to infect BgRES. We also observed that SmRES-2 is more capable to be transformed into SpI inside BgRES.

According to Theron et al. (15) the snail–schistosome compatibility results from a combination of changes and a balance in recognition and effector mechanisms. These changes and mechanisms depend on the genotypic diversity in the host and parasite isolates, and their sum determines the success or failure of the infection. Moreover, the compatibility polymorphism that exists at inter-strain level is also present at intra-strain level, between individual hosts/parasites of the same strain. A snail population is not homogenous regarding its susceptibility to schistosome. Each individual presents the ability to recognize and react against all miracidia (un-susceptibility) or solely to a part of the phenotypic diversity of a parasite strain (susceptibility). From our perspective, we are intrigued whether BgBAR modulated SmRES up to SmRES-2 by epigenetics mechanisms. After those 2 years, SmRES had to deal with BgBAR immune factors and in order to evade it; the strain changed its own establishment mechanisms and turned more infective than before. The establishment process is crucial and lasts 72 h after the begin of the infection. If the host immune defense system is effective, the parasite will be encapsulated, and the infection is ended. It is known that *B. glabrata* has a sophisticated immune system which allows it to recognize and remember prior *S. mansoni* infections, and shift their immune response from cellular to humoral (23).

We observed a huge difference on a unique intermediate host profile when it was facing the same parasite strain (BgRES/SmRES and BgRES/SmRES-2), considering the time SmRES-2 was interacting in an allopatric combination. These findings suggest that the success or failure of an infection does not depends only on the snail's susceptibility/resistance status, but rather on the match or mismatch status of hosts and parasites phenotypes (18). Furthermore, the compatible/incompatible status of a specific *B. glabrata/S. mansoni* interaction might reflects a sum of phenomenon, which plays a balance of multi molecular determinants. According to Mitta et al. (9) this phenomenon could be classified into two categories, the first corresponds to effector/anti-effector system of the host and the parasite, and the involved molecular determinants tend to induce resistance. The second corresponds to immune receptors and antigens, whose intraindividual diversification and polymorphisms could favor the match or mismatch status of host and parasite phenotype. Moreover, the issue became more complex when we also consider the environmental factors that are already known to influence the compatibility between host and parasite. Indeed, successive exposures of *B. glabrata* to schistosome can change the phenotype of both partners, thereby altering their compatibility.

Our future perspective is to understand this phenomenon involving BgRES and SmRES/SmRES-2, considering “omics” approaches, such as proteomics and transcriptomics. Moreover, we intend to also investigate other strains stocks from LABMAL. At this moment, we are carrying out genetic population's surveys on the 81 populations of *Biomphalaria* spp.

Finally, these studies will be useful to base laboratory studies and could give a general idea on how we must face the laboratory studies on the diversity of parasite and host strains. Later, these studies may also help to provide detailed information on the most important molecular determinants related to the host–parasite compatibility polymorphism. To conclude, we must emphasize that laboratory strains are useful tools to study compatibility. Despite the fact that they are not representative of their corresponding field populations, they may contribute to identify, in wild populations, molecular markers that were previously found in inbred snails' and trematode' populations.

## Author Contributions

ML, LM, RA, ST, and JP: conceptualization; ML, LM, RA, ST, and JP: data curation; ML, LM, JP, and RA: formal analysis; JaP and ST: funding acquisition; ML, LM, JP, and RA: investigation; ML, LM, JP, and RA: methodology; ML, LM, JaP, RA, and ST: project administration; ML, LM, RA, JaP, and ST: writing ± original draft.

### Conflict of Interest Statement

The authors declare that the research was conducted in the absence of any commercial or financial relationships that could be construed as a potential conflict of interest.

## References

[1] WHO Schistosomiasis. Fact Sheet No. 115. Geneva: World Health Organisation (2015).

[2] Steinmann P Keiser J Bos R Tanner M Utzinger J. Schistosomiasis and water resources development: systematic review, meta-analysis, and estimates of people at risk. Lancet Infect Dis. (2006) 6:411–25. 10.1016/S1473-3099(06)70521-7 16790382

[3] Tennessen JA Theron A Marine M Yeh JY Rognon A Blouin MS. Hyperdiverse gene cluster in snail host conveys resistance to human schistosome parasites. PLoS Genet. (2015) 11:e1005067. 10.1371/journal.pgen.1005067 25775214 PMC4361660

[4] Combes C Parasitism: The Ecology and Evolution of Intimate Interactions. Chicago, IL: Chicago University Press (2001).

[5] Shrivastava J Gower CM Balolong E Wang TP Qian BZ Webster JP. Population genetics of multi-host parasites—the case for molecular epidemiological studies of Schistosoma japonicum using larval stages from naturally infected hosts. Parasitology. (2005) 131:617–26. 10.1017/S0031182005008413 16255820

[6] Sorensen RE Rodrigues NB Oliveira G Romanha AJ Minchella DJ. Genetic filtering and optimal sampling of Schistosoma mansoni populations. Parasitology. (2006) 133:443–51. 10.1017/S0031182006000552 16817994

[7] Grech K Watt K Read AF. Host–parasite interactions for virulence and resistance in a malaria model system. J Evol Biol. (2006) 19:1620–30. 10.1111/j.1420-9101.2006.01116.x 16910991

[8] Zavodna M Sandland GJ Minchella DJ. Effects of intermediate host genetic background on parasite transmission dynamics: a case study using *Schistosoma mansoni*. Exp Parasitol. (2008) 120:57–61. 10.1016/j.exppara.2008.04.021 18538767 PMC2568033

[9] Mitta G Gourbal B Grunau C Knight M Bridger JM Théron A. The compatibility between *Biomphalaria glabrata* snails and *Schistosoma mansoni*: an increasingly complex puzzle. Adv Parasitol. (2017) 97:111–45. 10.1016/bs.apar.2016.08.006 28325369

[10] Galinier R Roger E Mone Y Duval D Portet A Pinaud S. A multistrain approach to studying the mechanisms underlying compatibility in the interaction between *Biomphalaria glabrata* and *Schistosoma mansoni*. PLoS Negl Trop Dis. (2017) 11:e0005398. 10.1371/journal.pntd.0005398 28253264 PMC5349689

[11] Portet A Pinaud S Tetreau G Galinier R Cosseau C Durval D. Integrated multi-omic analyses in *Biomphalaria-Schistosoma* dialogue reveal the immunobiological significance of FREP-SmPoMuc interaction. Dev Comp Immunol. (2017) 75:16–27. 10.1016/j.dci.2017.02.025 28257854

[12] Basch PF. Intermediate host specificity in *Schistosoma mansoni*. Exp Parasitol. (1976) 39:150–69. 10.1016/0014-4894(76)90022-9 767127

[13] Theron A Pages JR Rognon A. Schistosoma mansoni: distribution patterns of miracidia among *Biomphalaria glabrata* snail as related to host susceptibility and sporocyst regulatory processes. Exp Parasitol. (1997) 85:1–9. 10.1006/expr.1996.4106 9024196

[14] Theron A Coustau C Rognon A Gourbiere S Blouin MS. Effects of laboratory culture on compatibility between snails and schistosomes. Parasitology. (2008) 135:1179–88. 10.1017/S0031182008004745 18700994

[15] Theron A Rognon A Gourbal B Mitta G. Multiparasite host susceptibility and multihost parasite infectivity: a new approach of the *Biomphalaria glabrata/Schistosoma mansoni* compatibility polymorphism. Infect. Genet. Evol. (2014) 26:80–8. 10.1016/j.meegid.2014.04.025 24837670

[16] Lie RJ Heyneman D Richards CS. Specificity of natural resistance to trematode infections in *Biomphalaria glabrata*. Int J Parasitol. (1979) 9:529–31. 10.1016/0020-7519(79)90008-0 541166

[17] Webster JP Davies CM. Coevolution and compatibility in the snail-schsitosome system. Parasitology. (2001) 123:S41–56. 10.1017/S0031182001008071 11769292

[18] Theron A Coustau C Are *Biomphalaria glabrata* resistant to *Schistosoma mansoni*? Helminthology. (2005) 79:187–91. 10.1079/JOH2005299 16153311

[19] Neves BJ Dantas RF Senger MR Valente WCG Rezende-Neto JM Chaves WT The antidepressant drug paroxetine as a new lead candidate in schistosome drug discovery. Med Chem Commun. (2016) 7:1176–82. 10.1039/c5md00596e

[20] Silva ICA Vitor Coutinho Carneiro VC Vicentino ARR Aguilera EA Mohana-Borges R Thiengo S The distinct C-terminal acidic domains of HMGB proteins are functionally relevant in *Schistosoma mansoni*. Int J Parasitol. (2016) 46:253–62. 10.1016/j.ijpara.2015.12.007 26820302

[21] Allienne JF Theron A Gourbal B Recovery of primary sporocysts *in vivo* in the *Schistosoma mansoni/Biomphalaria glabrata* model using a simple fixation method suitable for extraction of genomic DNA and RNA. *Exp Parasitol*. (2011) 129:11–6. 10.1016/j.exppara.2011.06.003 21726555

[22] Moné Y Ribou AC Cosseau C Duval D Theron A Mitta G. An example of molecular co-evolution: reactive oxygen species (ROS) and ROS scavenger levels in *Schistosoma mansoni/Biomphalaria glabrata* interactions. Int J Parasitol. (2011) 41:721–30. 10.1016/j.ijpara.2011.01.007 21329695

[23] Pinaud S Portela J Durval D Nowacki FC Olive MA Allienne JF A shift from cellular to humoral response contributes to innate immune memory in vector snail *Biomphalaria glabrata*. PLoS Pathog. (2016) 12:e1005361 10.1371/journal.ppat.1005361 26735307 PMC4703209

