# Supplementary material for: Compatibility Polymorphism Based on Long-Term Host-Parasite Relationships: Cross Talking Between Biomphalaria glabrata and the Trematode Schistosoma mansoni From Endemic Areas in Brazil
Source: Front Immunol. 2019 Apr 9;10:328. doi: 10.3389/fimmu.2019.00328 (PMC6467164; doi:10.3389/fimmu.2019.00328)
Supplement: Supplementary file 2 [file Table_1.docx]

**(Supplementary material) Table 1-** The intensity of the infection of *Schistosoma mansoni* to *Biomphalaria glabrata* strains considering the miracidial doses and the SpI development. Variance analysis using ANOVA and Tukey post hoc test (P < 0.05) (R version 3.4.4). Means that are significantly different are indicated with an asterisk (*).

**The table shows only the pairwise comparisons that showed significant difference in the Tukey post hoc test P < 0.05.**

| ***Schistosoma mansoni* - RES** | | |  | | |
| --- | --- | --- | --- | --- | --- |
| **Comparison** | **t value** | **P-value** | **Comparison** | **t value** | **P-value** |
| BAR 1 Mi vs BAR 15 Mi | -3.838 | 0.0110 * | RES 1 Mi vs RES 15 Mi | -6.043 | <0.01 *** |
| RES 1 Mi vs BAR 5 Mi | -3.838 | 0.0109 * | RES 5 Mi vs RES 15 Mi | -4.700 | <0.01 *** |
| RES 1 Mi vs BAR 10 Mi | -4.349 | <0.01 ** | TEO 1 Mi vs RES 15 Mi | -5.148 | <0.01 *** |
| RES 1 Mi vs BAR 15 Mi | -5.117 | <0.01 *** | TEO 5 Mi vs RES 15 Mi | -4.476 | <0.01 *** |
| RES 5 Mi vs BAR 10 Mi | -3.582 | 0.0240 * | TEO 10 Mi vs RES 15 Mi | -3.357 | 0.0475 * |
| RES 5 Mi vs BAR 15 Mi | -4.349 | <0.01 ** | TEO 15 Mivs RES 1 Mi | 3.357 | 0.0481 * |
| RES 10 Mi vs BAR 10 Mi | -3.326 | 0.0518 . | RES 1 Mi vs RES 15 Mi | -6.043 | <0.01 *** |
| RES 10 Mi vs BAR 15 Mi | -4.093 | <0.01 ** | ***Schistosoma mansoni* - TEO** | | |
| TEO 1 Mi vs BAR 5 Mi | -3.582 | 0.0245 * | BAR 1 Mi vs BAR 15 Mi | -3.838 | 0.0191 * |
| TEO 1 Mi vs BAR 10 Mi | -4.093 | <0.01 ** | RES 1 Mi vs BAR 5 Mi | -3.838 | 0.0183 * |
| TEO 1 Mi vs BAR 15 Mi | -4.861 | <0.01 *** | RES 1 Mi vs BAR 10 Mi | -4.349 | 0.00189 ** |
| TEO 5 Mi vs BAR 5 Mi | -3.326 | 0.0520 . | RES 1 Mi vs BAR 15 Mi | -5.117 | < 0.001 *** |
| TEO 5 Mi vs BAR 10 Mi | -3.838 | 0.0113 * | RES 5 Mi vs BAR 10 Mi | -3.582 | 0.02438 * |
| TEO 5 Mi vs BAR 15 Mi | -4.605 | <0.01 *** | RES 5 Mi vs BAR 15 Mi | -4.349 | 0.00173 ** |
| TEO 10 Mi vs BAR 10 Mi | -3.582 | 0.0240 * | RES 10 Mi vs BAR 10 Mi | -3.326 | 0.05205 . |
| TEO 10 Mi vs BAR 15 Mi | -4.349 | <0.01 ** | RES 10 Mi vs BAR 15 Mi | -4.093 | 0.00470 ** |
| TEO 15 Mivs BAR 15 Mi | -3.838 | 0.0109 * | TEO 1 Mi vs BAR 5 Mi | -3.582 | 0.02475 * |
| ***Schistosoma mansoni* – RES-2** | | | TEO 1 Mi vs BAR 10 Mi | -4.093 | 0.00447 ** |
| RES 1 Mi vs BAR 15 Mi | -3.133 | 0.0873 * | TEO 1 Mi vs BAR 15 Mi | -4.861 | < 0.001 *** |
| RES 15 Mivs BAR 1 Mi | 4.924 | <0.01 *** | TEO 5 Mi vs BAR 5 Mi | -3.326 | 0.05216 * |
| RES 15 Mivs BAR 5 Mi | 4.252 | <0.01 ** | TEO 5 Mi vs BAR 10 Mi | -3.838 | 0.0155 * |
| RES 15 Mivs BAR 10 Mi | 3.133 | 0.0876 . | TEO 5 Mi vs BAR 15 Mi | -4.605 | < 0.001 *** |
| RES 1 Mi vs RES 10 Mi | -3.805 | 0.0120 * | TEO 10 Mi vs BAR 10 Mi | -3.582 | 0.02440 * |
